# Carboxypeptidase E/NFα1: A New Neurotrophic Factor against Oxidative Stress-Induced Apoptotic Cell Death Mediated by ERK and PI3-K/AKT Pathways

**DOI:** 10.1371/journal.pone.0071578

**Published:** 2013-08-15

**Authors:** Yong Cheng, Niamh X. Cawley, Y. Peng Loh

**Affiliations:** Section on Cellular Neurobiology, Program on Developmental Neuroscience, *Eunice Kennedy Shriver* National Institute of Child Health and Human Development, National Institutes of Health, Bethesda, Maryland, United States of America; University of Louisville, United States of America

## Abstract

Mice lacking Carboxypeptidase E (CPE) exhibit degeneration of hippocampal neurons caused by stress at weaning while over-expression of CPE in hippocampal neurons protect them against hydrogen peroxide-induced cell death. Here we demonstrate that CPE acts as an extracellular trophic factor to protect neurons. Rat hippocampal neurons pretreated with purified CPE protected the cells against hydrogen peroxide-, staurosporine- and glutamate-induced cell death. This protection was observed even when hippocampal neurons were treated with an enzymatically inactive mutant CPE or with CPE in the presence of its inhibitor, GEMSA. Purified CPE added to the culture medium rescued CPE knock-out hippocampal neurons from cell death. Both ERK and AKT were phosphorylated within 15 min after CPE treatment of hippocampal neurons and, using specific inhibitors, both signaling pathways were shown to be required for the neuroprotective effect. The expression of the anti-apoptotic protein, B-cell lymphoma 2 (BCL-2), was up-regulated after hippocampal neurons were treated with CPE. Furthermore, hydrogen peroxide induced down-regulation of BCL-2 protein and subsequent activation of caspase-3 were inhibited by CPE treatment. Thus, this study has identified CPE as a new neurotrophic factor that can protect neurons against degeneration through the activation of ERK and AKT signaling pathways to up-regulate expression of BCL-2.

## Introduction

Neurological diseases such as Alzheimer’s disease and Parkinson’s disease, as well as various types of stress including excess glucocorticoids, glutamate neurotoxicity and ischemia lead to neuronal cell death [1], [2], [3], [4]. Recent studies have suggested that carboxypeptidase E (CPE) is involved in neuroprotection [5]. CPE was first discovered as an enkephalin convertase in 1982 [6], [7] and was subsequently found to be the enzyme that cleaves the C-terminally extended basic residues from peptide intermediates in endocrine cells and neuropeptides in peptidergic neurons (for review see [8]). Since then, various non-enzymatic roles of CPE have been found. CPE acts as a sorting receptor to target proneuropeptides and pro-brain-derived neurotrophic factor (pro-BDNF) to the regulated secretory pathway [9], [10]. Additionally, the cytoplasmic tail of CPE mediates BDNF vesicle transport [11] and synaptic vesicle localization to the nerve terminal preactive zone [12].

The idea of the involvement of CPE in neuroprotection evolved from an animal model of global ischemia [13]. Neurons from the CA3 region of the hippocampus survived after transient global ischemia and correlated with greater and more sustained increased expression of CPE. By contrast, neurons from the CA1 region of the hippocampus, which were more susceptible to degeneration, showed only a transient up-regulation of CPE. In another study, while expression of CPE was up-regulated in neurons in the hippocampal CA3 region and survived after focal cerebral ischemia in wild-type (WT) mice, these neurons exhibited cell death in *Cpe^fat/fat^* mutant mice lacking CPE [14]. Mice subjected to mild chronic restraint stress also showed up-regulation of CPE in the hippocampus and increased expression of the anti-apoptotic protein, BCL-2; but this did not occur in CPE knock-out (CPE-KO) mice devoid of CPE, and in fact showed decreased BCL-2 levels [15], [16]. Additionally, CPE-KO, but not WT mice exhibited neurodegeneration in the CA3 region of the hippocampus after weaning stress, which includes maternal separation, tail clipping for genotyping and ear tagging [17], [18]. Studies also showed that postnatal day 6 cultured cerebellar granule neurons from *Cpe* ^+/−^ mice with reduced CPE expression exhibited greater cell death after K^+^ deprivation (5 mM) compared with *Cpe* ^+/+^ mice [5]. Direct evidence of a neuroprotective role of CPE came from the study showing that transduction of CPE into primary cultured hippocampal neurons protected them against oxidative stress-induced cell death [17]. However, the mechanism of the neuroprotective action of CPE remained elusive. It was unclear whether CPE could play a neuroprotective role acting intracellularly, since it is made in the rough endoplasmic reticulum and packaged inside secretory vesicles; or rather, it could act extracellularly since it is secreted from neurons [19].

Indeed, recent studies indicate that CPE can function extracellularly. CPE forms a complex with Wnt 3a ligand and frizzled receptor to act as a negative regulator of the Wnt signaling pathway in HEK293 cells [20]. In glioma cells, extracellular CPE decreased their migration and increased their proliferation [21]. Also, extracellular CPE has been shown to be a negative regulator of proliferation of adult neural stem cells (neurospheres) [22]. However, how CPE brings about these effects is not understood. In our present study, we investigated the extracellular role and mechanism of action of CPE in neuroprotection and cell survival. We demonstrate for the first time that CPE acts extracellularly through activation of ERK and AKT signaling pathways to up-regulate expression of the anti-apoptotic protein BCL-2 to mediate neuroprotection of neurons during stress.

## Materials and Methods

### Ethics Statement

All animal studies described herein were done with the approval of the Animal Care and Use Committee, NICHD, NIH. To reduce the stress to the animals, the animals were euthanized by CO_2_ inhalation at a 30% chamber fill rate until a lack of respiration and faded eye color was observed for at least 1 min. The animals were then immediately decapitated.

### Reagents

LY294002 and U0126 were purchased from Cell Signaling; K252a, PD166285 and GEMSA were purchased from Sigma. Adenoviral vectors (Type 5 (dE1/E3)), carrying the cDNA of WT CPE or CPE(E300Q) mutant, were custom made by Vector Biolabs, Philadelphia, PA. The adenoviral vector carrying the LacZ cDNA was also purchased from Vector Biolabs and used as a negative control. The vectors were validated by transduction of COS7 cells and analysis of the conditioned media by Western blot; similar levels of WT and E300Q CPE were detected (data not shown). The conditioned media were further assayed for CPE enzymatic activity using an adrenocorticotropin (ACTH) peptide intermediate (ACTH(1–17); ACTH_1–14_-Lys_15_-Lys_16_-Arg_17_) as a substrate, purchased from Bachem, Torrance, CA. Only the conditioned medium containing WT CPE generated ACTH(1–16) and ACTH(1–15) in a cobalt inducible manner whereas the E300Q mutant was inactive as expected [23] (data not shown).

### Animals

Pregnant rats were purchased from Taconic Farms, Inc., Derwood, MD. CPE-KO mice (on a C57BL6 background, backcrossed >10 generations from the original C57BL6/SV129 strain) [24] and wild type (WT) littermates, were raised in our animal facility. All animals were given food and water *ad libitum* in a humidity and temperature controlled room under a 12 h light:dark cycle.

### Recombinant Carboxypeptidase E

Purified recombinant WT CPE was custom generated by Creative Biolabs, Shirley, NY. Briefly, a mammalian expression vector containing the full length cDNA of WT mouse CPE, produced in our laboratory, was used as a template for sub-cloning into a proprietary expression vector by Creative Biolabs. Six histidines were added to the extreme C-terminus of CPE which was followed by a stop codon. Using this plasmid, CPE was expressed in HEK293 cells after transient transfection and purified from the conditioned medium using divalent metal chelating affinity chromatography. The column eluate was desalted by diafiltration with sterile PBS, pH 7.2, to remove the imidazole, aliquoted and frozen at −80°C until use. Analysis of the protein by 1) SDS PAGE and Coomassie Blue staining confirmed an apparent homogeneous preparation of CPE, 2) Western blot showed one major band at the correct size of CPE (a very faint immunoreactive band was occasionally seen at ∼20 kDa and is a C-terminal containing breakdown fragment of CPE) and 3) Enzyme activity, using ACTH(1–17) as substrate, demonstrated that the CPE was active in a dose dependent manner. In addition, all activity was eliminated in the presence of GEMSA (2-guanidinoethylmercaptosuccinic acid), a potent specific inhibitor of CPE [25] (Fig. S1).

### Primary Neuronal Culture

#### Rat hippocampal neurons

E18 embryos were obtained from rats and their brains removed. Hippocampal neuronal cultures were prepared as described previously with modifications [26]. Briefly, the hippocampus was dissected and digested by 2 ml papain (2 mg/ml) for 30 min at 37°C, which was then inactivated by 3 ml of 10% FBS. The tissue was triturated by a pipette to make a homogenous mixture which was then passed through a cell strainer to remove undissociated tissue. The cells were then centrifuged for 5 min at 1500×g, and the supernatant discarded. The cell pellet was resuspended in DMEM containing 1X antibiotics (Penicillin-Streptomycin) and 5% FBS. The cells were then plated on poly-L-lysine (Sigma) coated plates at a density of 1×10^6^ cells/ml. The medium was replaced by Neurobasal medium with 2% B27 (Invitrogen) after plating over-night.

#### Mouse hippocampal neurons

A litter of embryonic day 17 (E17) pups, derived from mating two heterozygote (*Cpe^+/−^*) mice, were harvested. Embryonic hippocampal neurons were isolated from the embryos as described previously for cortical neurons, with modifications [27]. Cells from each embryo were handled individually and mechanically dissociated and plated in separate poly-L-lysine-treated dishes. Cells belonging to WT or *Cpe^−/−^* (CPE knockout (KO)) pups were identified after genotyping [24]. The cells were grown in culture medium (DMEM supplemented with 10% FBS and antibiotics as indicated above). The next day, the medium was replaced with Neurobasal medium supplemented with 2% B27 and purified CPE (0.4 µM), where indicated. The media was replaced twice per week with fresh media containing new CPE. The cells were analyzed by the TUNEL assay (see below) after 2 weeks in culture.

### Treatment of Hippocampal Neurons with Conditioned Media, Recombinant CPE, H_2_O_2_, Staurosporine (STS) and Glutamate

Primary cultured hippocampal neurons (5–7 DIV) were transduced with adenovirus carrying LacZ, WT CPE or E300Q constructs (50 MOI) for 16 h, then washed with Hank’s balanced salt solutions and incubated with fresh neurobasal medium. After 24 h, the media were collected. These conditioned media were used to incubate new primary cultured hippocampal neurons in the presence or absence of 100 µM H_2_O_2._ Cell cytotoxicity was tested by the LDH release assay after 24 h of treatment. In other experiments, primary hippocampal neurons were incubated with purified recombinant CPE at various concentrations for 24 h. The neurons were then treated with 100 µM H_2_O_2_, 40 µM glutamate (Sigma) or 0.2 µM staurosporine (Sigma) for 24 h. Cell viability or cell cytotoxicity were then assayed by water-soluble tetrazolium (WST-1) or LDH assays (see below) or the cell lysates were analyzed by Western blot.

### Treatment of Hippocampal Neurons with CPE with or without ERK and AKT Inhibitors

Cultured hippocampal neurons were first incubated with 0.4 µM CPE for 0, 15, 30 and 60 min after which the cells were harvested and the corresponding lysates analyzed by Western blot for p-ERK and p-AKT. Subsequently, cultured hippocampal neurons were preincubated with or without the ERK inhibitor, U0126 (5 µM), or AKT inhibitor, LY294002 (10 µM), for 30 min after which 0.4 µM CPE was added, where indicated, and incubated for a further 30 min. The cells were then harvested and the corresponding cell lysates analyzed by Western blot for p-ERK and p-AKT. To determine if the ERK and AKT pathways are involved in the CPE dependent cell survival, cultured hippocampal neurons were preincubated with the ERK or AKT inhibitors for 30 min after which 0.4 µM CPE was added to the culture dishes, where indicated, and incubated for 24 h. The cells were then subjected to oxidative stress by the addition of H_2_O_2_ (100 µM) to the culture dishes for 24 h after which the cells were assayed for cell viability by the WST-1 assay (see below).

### WST-1 Assay for Cell Viability

The viability of the cells was determined by the WST-1 Cell Proliferation Reagent (Clonetech) assay in a 96 well plate according to the manufacturer’s protocol.

### LDH Release Assay for Cell Cytotoxicity

The cytotoxicity of cells after various treatments was evaluated by the extent of the release of LDH. This was achieved with a CytoTox 96 Non-Radioactive Cytotoxicity Assay kit according to the manufacturer’s instructions (Promega, Madison, WI).

### TUNEL Assay

An in situ cell death detection kit (TUNEL assay kit (Roche)) was used to stain the cells as described previously [28]. Briefly, primary cultured hippocampal neurons grown on slides were fixed and permeabilized after the various treatments. After staining by TUNEL and DAPI (4',6-diamidino-2-phenylindole), the images were recorded on a fluorescent microscope. The percentage of cell death was determined by the ratio of the number of TUNEL-positive cells over the total DAPI stained cells. At least 500 cells were counted in each well. The average of 6 wells was calculated as the percentage of neuronal cell death for the various treatments.

### Quantitative RT-PCR

Total RNA was extracted from the hippocampus or primary cultures of hippocampal neurons using Trizol (Invitrogen) and chloroform, and purified using the RNeasy mini kit (Qiagen) and quantified. First strand cDNAs were synthesized with 500 ng of RNA using Improm-II Reverse Transcription System (Promega). PCR amplification was carried out in the presence of 12.5 ng of cDNA template, 6.25 µl of Power SYBR green I Master Mix (Applied Biosystems), and 100 nM (18S rRNA) or 300 nM (Bcl-2) of forward and reverse primers, in 12.5 µl, in an ABI 7500 Sequence Detector (Applied Biosystems). The cycling conditions were: 10 min denaturation at 95°C and 40 cycles of DNA synthesis at 95°C for 15 s and 60°C for 1 min. Primer sequences for Bcl2 fwd: 5′-AAGCTGTCACAGAGGGGCTA-3′, rev: 5′-CAGGCTGGAAGGAGAAGATG-3′; for 18S-fwd: 5′-CTCTTAGCTGAGTGTCCCGC-3′, rev: 5′-CTGATCGTCTTCGAACCTCC-3′. Fluorescence signals were analyzed using SDS 1.9.1 software (Applied Biosystems). All qPCRs were performed in triplicates and were averaged to obtain the data point for each specimen. The relative amount of CPE mRNA was normalized to 18S rRNA.

### Western Blot

Soluble protein lysates of hippocampal neurons in culture were prepared by homogenizing the cells in T-protein extraction reagent (Pierce, Rockford, IL) supplemented with 1X Complete Inhibitor Cocktail (Roche) and centrifugation. Twenty µg of protein from the supernatants were analyzed by standard Western blotting procedures using nitrocellulose. Protein bands were visualized and quantified by the Odyssey infrared imaging system and software v2.1 (LI-COR Inc.). The protein expression level for each sample was normalized to β-actin. Monoclonal rabbit anti-cleaved active caspase-3 antibody (1∶3000), monoclonal mouse anti-p-AKT antibody (1∶3000), polyclonal rabbit anti-t-AKT antibody (1∶5000) and polyclonal rabbit anti-BCL-2 antibody (1∶3000) were from Cell Signaling. Monoclonal mouse anti-p-ERK antibody (1∶1000) and polyclonal rabbit anti-t-ERK antibody (1∶5000) were from Santa Cruz. Purified polyclonal rabbit anti-CPE antibody was generated in our laboratory.

### Statistical Analysis

Data were analyzed by Student’s *t-*test and one-way or two-way analysis of variance (ANOVA) followed by Tukey post-hoc multiple comparisons tests where noted. Significance was set at p<0.05.

## Results

### Secreted CPE Protected Primary Cultured Rat Hippocampal Neurons against Oxidative Stress

**Figure 1:**
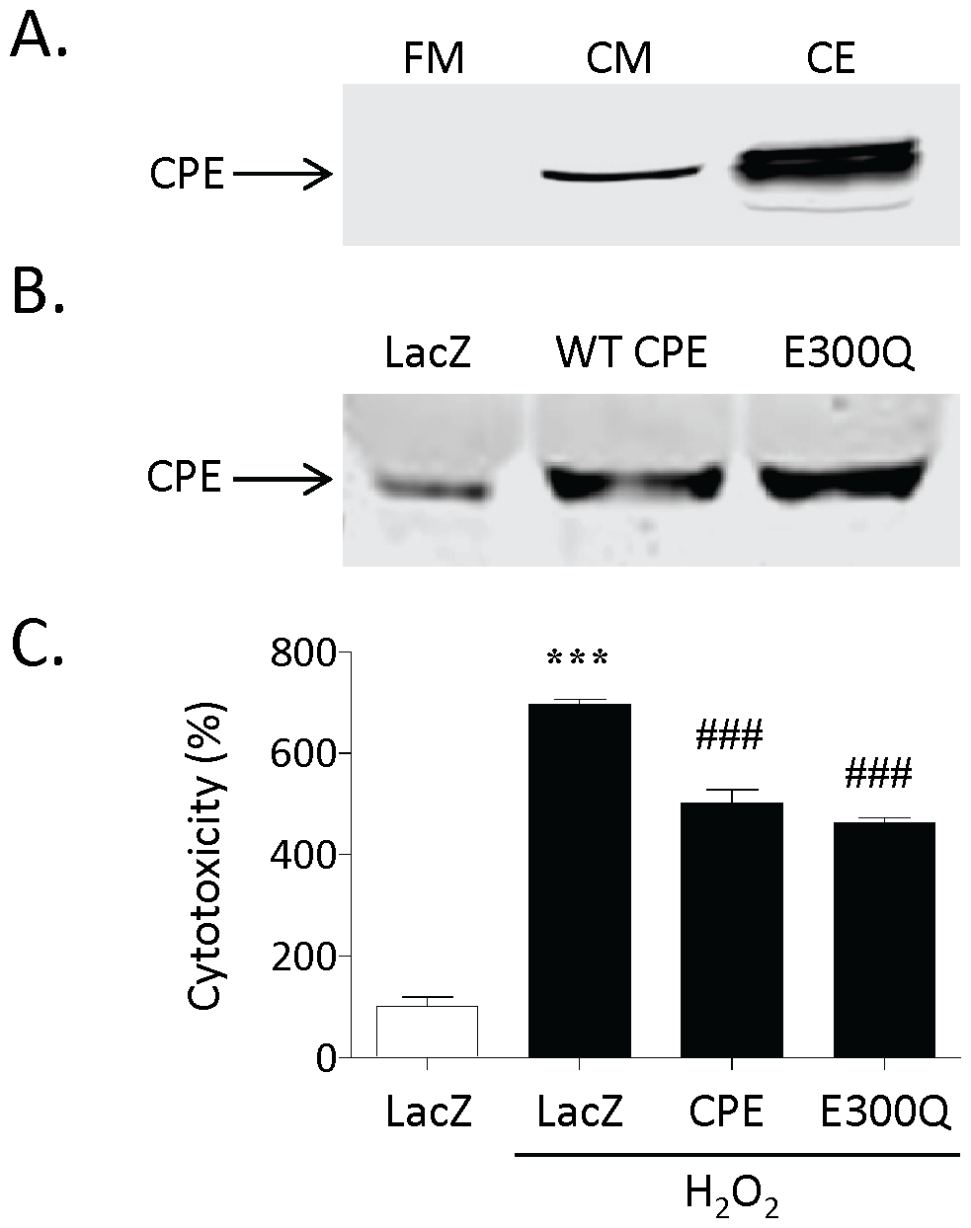
CPE containing conditioned medium protects against H_2_O_2_-induced cytotoxicity in primary cultured rat hippocampal neurons. **A)** Western blot showing the presence of endogenous CPE in the cell lysate (CE) and conditioned medium (CM) of hippocampal neurons. No signal was obtained from the non-conditioned or fresh medium (FM). **B)** Western blot showing CPE protein in conditioned medium from primary hippocampal neurons transduced with adenovirus harboring LacZ and WT or E300Q CPE constructs. **C)** Bar graphs showing LDH activity, as an indicator of cellular cytotoxicity, in the culture media of hippocampal neurons treated with and without H_2_O_2_. Note the increased cytotoxicity after H_2_O_2_ treatment (LacZ/H_2_O_2_) compared to control cells (LacZ) that was significantly attenuated by the presence of either WT (CPE/H_2_O_2_) or E300Q (E300Q/H_2_O_2_) CPE. Students t test, n = 4, ***p<0.001 compared to LacZ control; ^###^p<0.001, compared to LacZ/H_2_O_2_.

Our previous study showed that transduction of CPE into primary cultured hippocampal neurons protected them against H_2_O_2_ induced neurotoxicity [17]. To determine if CPE could play a neuroprotective role extracellularly, we first determined if endogenous CPE is secreted from hippocampal neurons. As shown in Fig. 1A, endogenous CPE was detected by Western blot in the conditioned medium of cultured hippocampal neurons.

We then collected conditioned medium from primary cultured hippocampal neurons transduced with adenoviral constructs over-expressing LacZ (control), WT CPE and CPE(E300Q). Western blots of the conditioned media showed that both forms of CPE were over-expressed and secreted from the neurons (Fig. 1B). When this conditioned media was incubated with new primary cultured hippocampal neurons, which were then challenged with H_2_O_2,_ the neurons exhibited less toxicity after H_2_O_2_ treatment compared to neurons pretreated with the control medium (Fig. 1C, n = 4, p<0.001). The results with the E300Q mutant suggest that the neuroprotective effect of CPE is independent of its enzymatic activity. This was further confirmed by experiments showing that addition of GEMSA, a specific and potent inhibitor of CPE enzymatic activity [25] did not affect the neuroprotective activity of WT CPE (Fig. S2).

### Purified Recombinant CPE is Neuroprotective in Primary Cultured Rat Hippocampal Neurons

**Figure 2:**
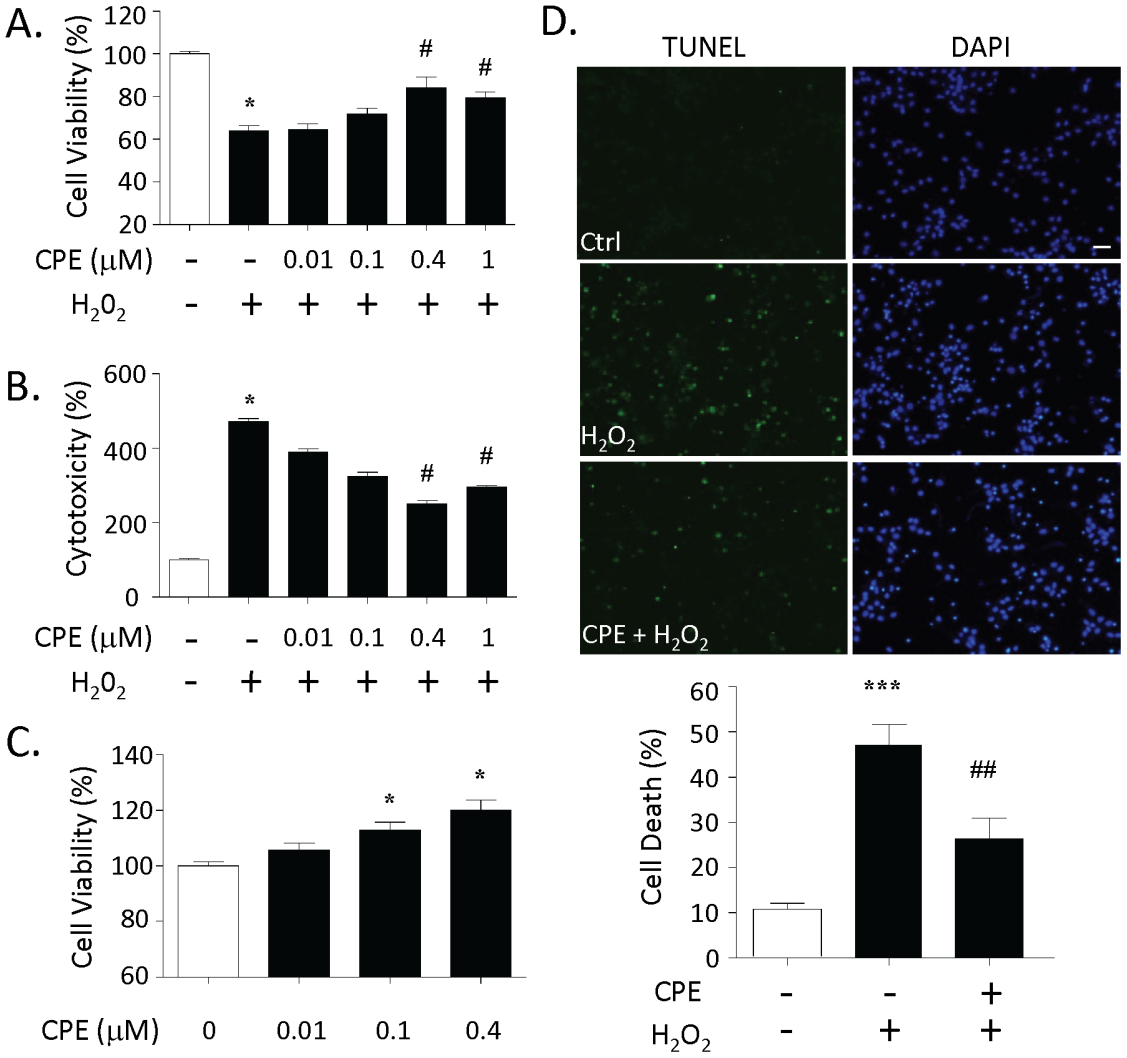
Purified recombinant CPE protein protects against H_2_O_2_-induced neurotoxicity in primary cultured rat hippocampal neurons. **A)** Bar graphs showing WST activity, indicative of cell viability, of hippocampal neurons treated with and without H_2_O_2_. Note the reduced cell viability after H_2_O_2_ treatment that was significantly attenuated by the pretreatment of the neurons with 0.4 µM and 1 µM purified CPE. **B)** Bar graphs showing LDH activity, as in figure 1, in the culture media of hippocampal neurons treated with and without H_2_O_2_. Note that the H_2_O_2_-induced cytotoxicity was significantly attenuated by the pretreatment of the neurons with 0.4 µM and 1 µM purified CPE. **C)** Bar graphs showing WST activity of hippocampal neurons treated with purified CPE. Note that cell viability increased significantly by treatment with 0.4 µM and 1 µM CPE. **D)** Photomicrographs of hippocampal neurons with and without H_2_O_2_ treatment stained by TUNEL (green) and DAPI (blue). Note that the number of dead cells (green) increased significantly after H_2_O_2_ treatment (middle, left panel) and that pretreatment with 0.4 µM CPE protected the neurons (bottom, left panel). The bar graph represents the quantification of the dead cells as a % of the total number of cells determined by the DAPI staining. 500 cells were counted in each of 6 different dishes generated from embryos from 2 rats. **A–C**, one way ANOVA with Tukey post-hoc test, n = 5, *p<0.05 when compared to control cells; ^#^p<0.05 compared to H_2_O_2_ treated only cells. **D**, Students t test, n = 6, ***p<0.001 compared to control cells; ^##^p<0.01 compared to H_2_O_2_ treated only cells. Bar = 100 µm.

To confirm that the neuroprotective effect of the conditioned medium came from CPE specifically, we used purified recombinant mouse CPE protein added to the culture media. As shown in Fig. 2A, we show that the cell viability of cultured hippocampal neurons decreased significantly after H_2_O_2_ treatment compared to the control group (n = 5, *p<0.05). However, the severity of this decrease in cell viability was significantly reduced when the neurons were pretreated with 0.4 µM and 1 µM CPE (n = 5, #p<0.05). Analysis of LDH release, as a measure of cytotoxicity, showed that H_2_O_2_ significantly increased cytotoxicity in the neurons compared to the control group (n = 5, * p<0.05), however, the severity of this cytotoxicity was significantly reduced by pretreatment of the neurons with 0.4 µM and 1 µM of CPE (n = 5, #p<0.05) (Fig. 2B). Moreover, we found that treatment with CPE alone increased the cell viability at 0.1 µM and 0.4 µM (n = 5, *p<0.05) after 24 h of treatment (Fig. 2C). A similar neuroprotective effect of CPE on staurosporine- (STS) and glutamate-induced neurotoxicity was also seen (Fig. S3).

Since the above results indicated that the neuroprotective effects of CPE peaks at 0.4 µM, this concentration was used in all subsequent experiments. The TUNEL assay was used to further confirm the protective effects of CPE in the cultured hippocampal neurons. Fig. 2D shows that the number of dead hippocampal neurons increased significantly after treatment with H_2_O_2_ compared to the control group (n = 6, p<0.001); however, the number of dead cells was significantly reduced by pretreatment of the neurons with 0.4 µM CPE (n = 6, p<0.01).

### Extracellular CPE Rescued Cell Death of Hippocampal Neurons from CPE Knockout Mice

**Figure 3:**
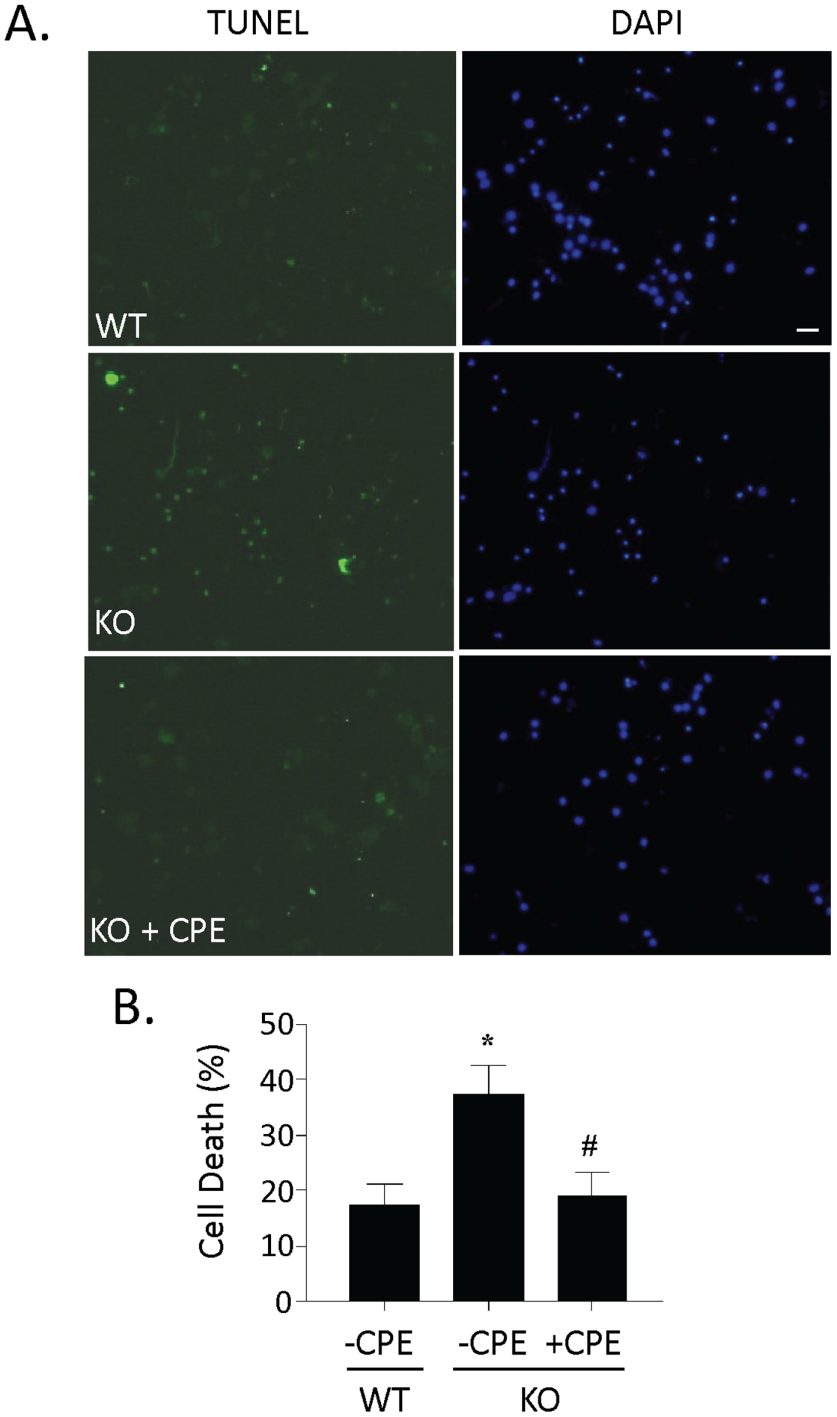
Purified recombinant CPE rescues cell death in CPE knock-out mouse hippocampal neurons. **A)** Photomicrographs of 2 week cultures of hippocampal neurons derived from WT and CPE-KO embryos stained by TUNEL (green) and DAPI (blue). Note the higher number of dead cells (green) in the CPE-KO culture (KO) compared to the WT culture (WT). Culturing the CPE-KO neurons in the presence of 0.4 µM CPE significantly reduced the number of dead cells (KO+CPE). **B)** The bar graph represents the quantification of the dead cells as a % of the total number of cells determined by the DAPI staining. Students t test, n = 4, *p<0.05 compared to WT neurons; ^#^p<0.05 compared to KO neurons not treated with CPE. Bar = 100 µm.

Hippocampal neurons from E17 WT and CPE-KO mouse embryos were cultured for two weeks. As shown by the TUNEL assay in Fig. 3, primary cultured hippocampal neurons devoid of CPE exhibited significantly higher cell death compared to WT neurons (n = 4, *p<0.05). Adding purified recombinant CPE to the CPE-KO neurons during the 2 weeks in culture decreased the amount of cell death compared to untreated CPE-KO neurons (n = 4, #p<0.05).

### CPE Activated ERK and AKT Pathways to Protect Cultured Rat Hippocampal Neurons

**Figure 4:**
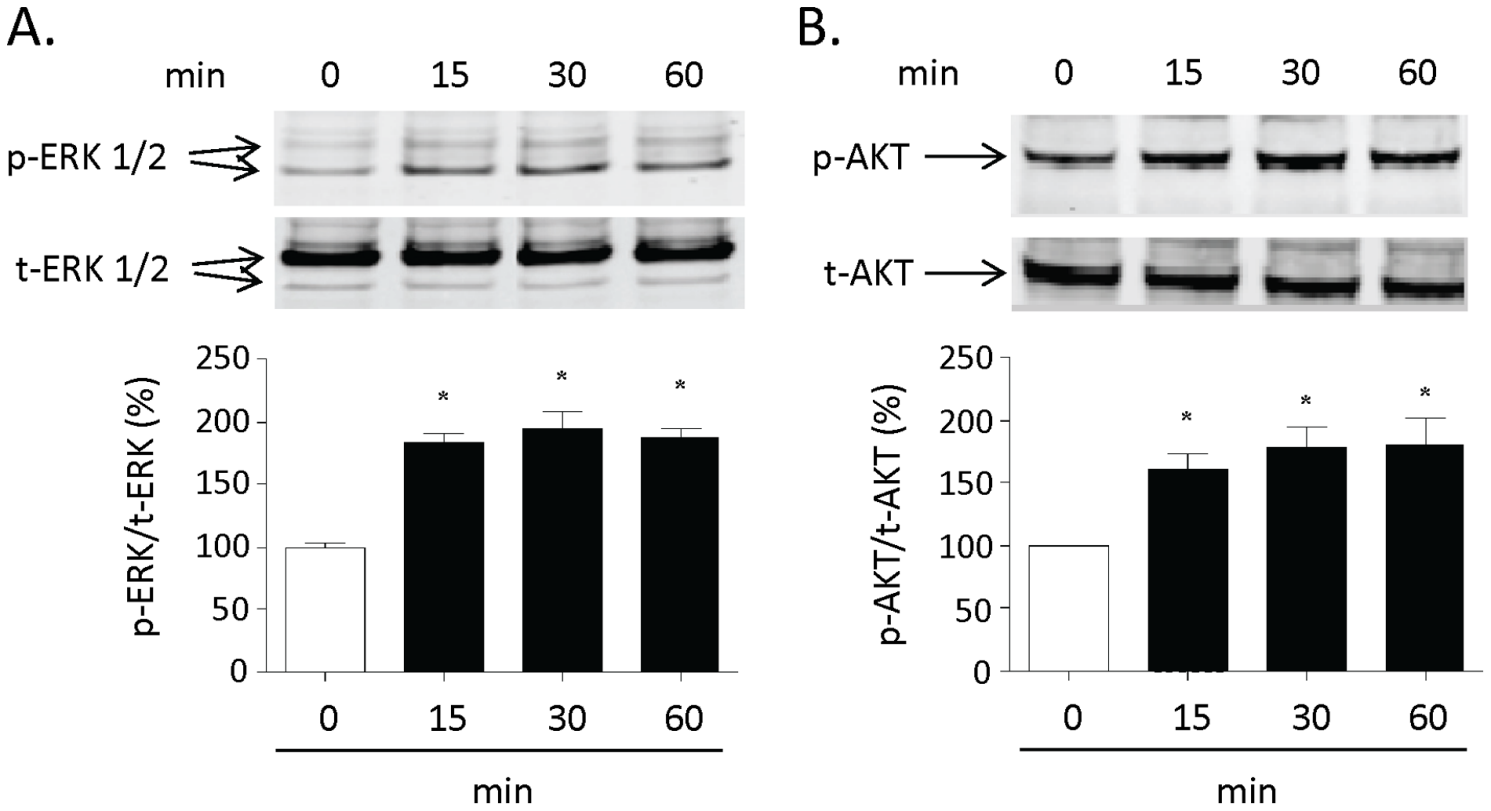
CPE activates ERK and AKT signaling pathways in primary cultured rat hippocampal neurons. Top panels: Representative Western blot analyses of hippocampal neuron lysates showing p-ERK 1/2 (**A**) and p-AKT (**B**) after 0, 15, 30 and 60 min treatment with 0.4 µM CPE. Total ERK (t-ERK 1/2) and total AKT (t-AKT) were also analyzed and served as internal controls. Bottom panels: Bar graphs showing the quantification of the p-ERK 1/2 (**A**) and p-AKT (**B**) signals normalized to t-ERK 1/2 and t-AKT, respectively. Note the increase in p-ERK and p-AKT after 15, 30 and 60 min treatment with CPE. Statistical analysis for **A** and **B** by one way ANOVA with Tukey post-hoc test, n = 4, *p<0.05 when compared to untreated control cells.

Treatment of hippocampal neurons with purified CPE for 15, 30 and 60 min all resulted in significantly increased phosphorylation of ERK compared to the control group (Fig. 4A, n = 4, *p<0.05 for all time points). A similar increase in AKT phosphorylation, compared to the control group, was also obtained (Fig. 4B, n = 4, *p<0.05 for all time points). Thus both ERK and AKT signaling pathways are activated by treatment with CPE.

**Figure 5:**
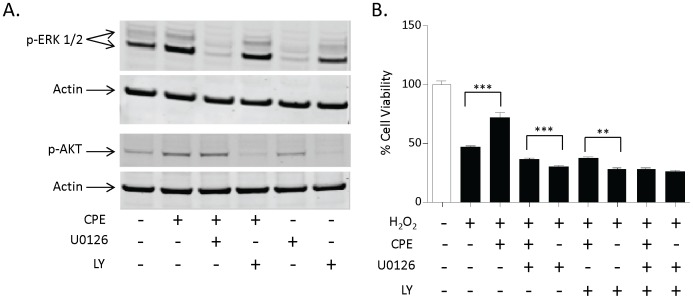
Both ERK and AKT signaling pathways are required for the neuroprotective effect of CPE. **A)** Western blot analysis showing that the CPE induced phosphorylation of ERK and AKT in primary cultured rat hippocampal neurons is blocked by U0126 and LY294002, respectively. **B)** Bar graphs showing WST activity, indicative of cell viability, of hippocampal neurons treated with and without H_2_O_2_ in the continued presence or absence of CPE and kinase inhibitors. Note that the neuroprotective effect of CPE is blocked by U0126 and LY294002 in primary cultured hippocampal neurons. Also note that maximal effects were observed when both inhibitors were used together, suggesting that ERK and AKT signaling pathways work in parallel to mediate the neuroprotective effect of CPE (t test, n = 5, ***p<0.001, **p<0.01).

To determine if the activation of ERK and AKT is required for the neuroprotective effects of CPE, we used the MEK inhibitor, U0126 and the PI3-K inhibitor, LY294002. Western blots showed that the CPE-induced phosphorylation of ERK was blocked by 5 µM U0126, but not 10 µM LY294002, while the CPE-induced phosphorylation of AKT was blocked by 10 µM LY294002 but not 5 µM U0126, demonstrating the specificity of the inhibitors (Fig. 5A). Next, the cultured hippocampal neurons were pretreated with vehicle, U0126, LY294002 or U0126 plus LY294002 for 30 min. Subsequently, CPE was added to the neurons for 24 h, and then the neurons were challenged with H_2_O_2_ for 24 h. As shown in Fig. 5B, CPE still had some neuroprotective effects against H_2_O_2_ in the presence of U0126 compared to the H_2_O_2_ plus U0126 group. Also, CPE still had some neuroprotective effects against H_2_O_2_ in the presence of LY294002 compared to the H_2_O_2_ plus LY294002 group. Interestingly, the neuroprotective effect of CPE against H_2_O_2_ was abolished in the presence of U0126 and LY294002 compared to the U0126+ LY294002+ H_2_O_2_ group, indicating that both ERK and AKT signaling pathways are mediating the neuroprotective effects of CPE.

### Neuroprotective Effect of CPE is Accompanied by the Increase of BCL-2 Expression and Inhibition of Caspase-3

**Figure 6:**
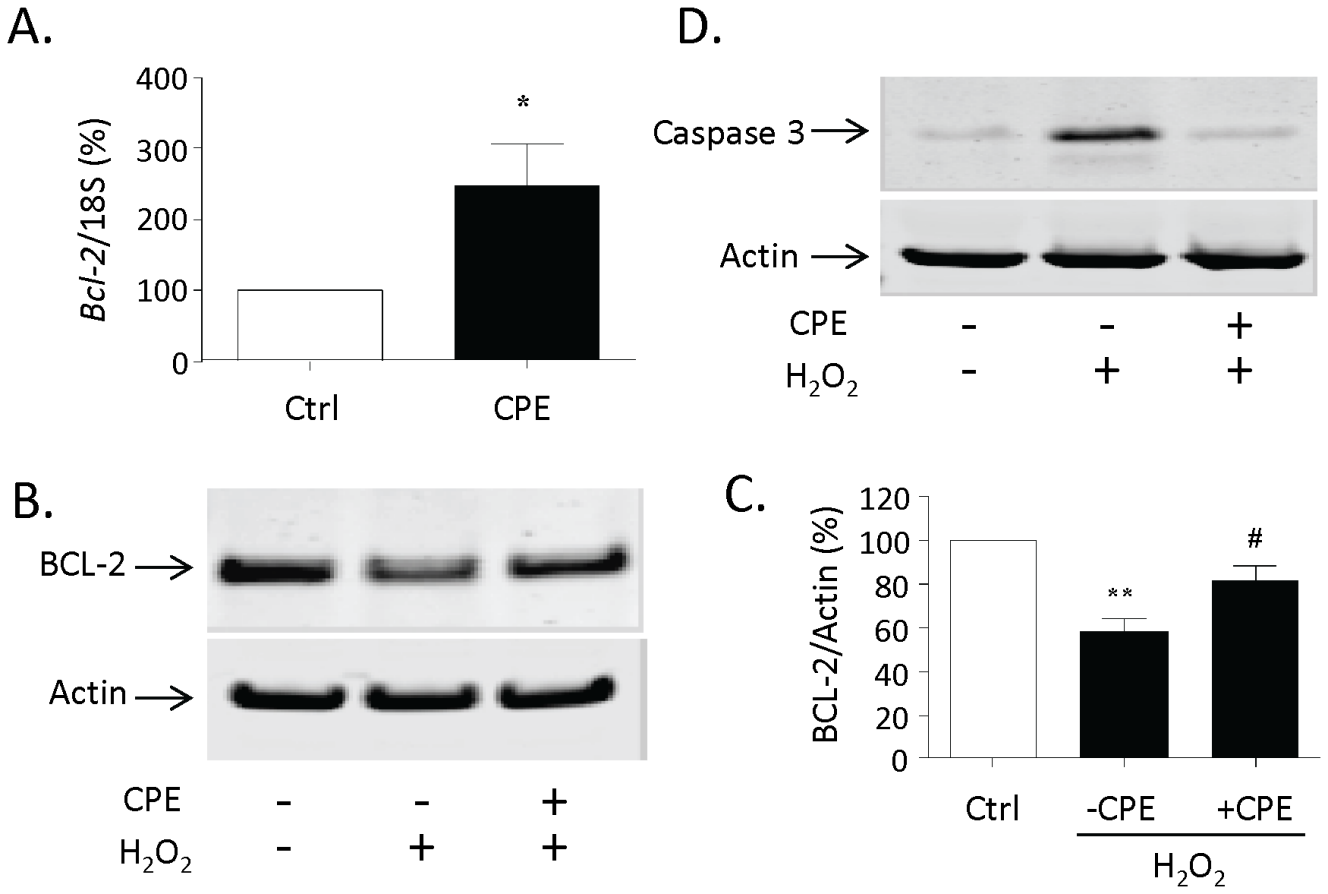
Neuroprotection by CPE involves BCL-2 and Caspase-3. **A)** Bar graph showing the quantification by qRT-PCR of *Bcl-2* mRNA in primary cultured rat hippocampal neurons after treatment with 0.4 µM CPE for 3 h. Data is normalized against 18S RNA and presented as a % compared to untreated control (Ctrl) cells, (t-test, n = 3, *p<0.05). **B)** Representative Western blot analysis of BCL-2 protein in primary cultured hippocampal neurons, pretreated with 0.4 µM CPE for 24 h and subsequently challenged or not with H_2_O_2_ for 24 h. Actin was also analyzed and served as an internal control for protein load. **C)** Bar graphs showing the quantification of BCL-2 protein normalized to Actin and expressed as a % compared to untreated control (Ctrl) cells. Note that CPE significantly inhibited the H_2_O_2_-induced decrease in BCL-2 protein in primary cultured hippocampal neurons, (t-test, n = 4, ** p<0.01 compared to Ctrl; ^#^ p<0.05 compared to H_2_O_2_ treated only cells) **D)** Western blot analysis of cleaved caspase-3 in primary cultured hippocampal neurons pretreated with 0.4 µM CPE for 24 h and subsequently challenged or not with H_2_O_2_ for 24 h. Note that the H_2_O_2_-induced activation of caspase-3 is blocked by CPE. Western blot is representative of 2 independent experiments.

To further investigate the mechanism, we analyzed the expression of the ERK and/or AKT down-stream target anti-apoptotic protein, BCL-2, in primary cultured hippocampal neurons after treatment for 3 h with CPE. Treated neurons were collected for RNA extraction and qRT-PCR. As shown in Fig. 6A, CPE significantly increased *Bcl-2* mRNA expression compared to the control group (n = 3, *p<0.05). We then analyzed the expression of BCL-2 protein after oxidative stress in primary cultured hippocampal neurons. Fig. 6B and C show that BCL-2 protein was decreased significantly in hippocampal neurons treated with H_2_O_2_ compared to the control group (n = 4, **p<0.01). However, pretreatment of the neurons with CPE significantly inhibited the H_2_O_2_-induced decrease of BCL-2 in the neurons compared to the H_2_O_2_ treated group (n = 4, #p<0.05). Moreover, we found that the activation of caspase-3 induced by H_2_O_2_ was blocked by pretreatment with CPE (Fig. 6D).

## Discussion

Previous studies have suggested that CPE, a prohormone processing enzyme has neuroprotective functions [5], [13], [14], [16], [17], [18]. However, in these and other studies where CPE is regulated by stress or disease [29], [30], [31], its presence was seen as primarily correlative and its function was unknown. In the current study we have investigated the potential mechanism of action of CPE in neuroprotection. Our findings identified a new role for CPE, as a neurotrophic factor that functions extracellularly to protect hippocampal neurons against oxidative stress-induced, staurosporine-induced and glutamate-induced apoptotic cell death and is consistent with the observation that CPE is secreted from neurons (Fig. 1A and [19]). In addition, this neuroprotective effect was also observed in cortical neurons subjected to oxidative stress (Fig. S4), indicating that it is not specific for hippocampal neurons only. Our studies also revealed that CPE is an important survival factor for embryonic neurons in culture, since CPE-KO mouse embryonic hippocampal neurons devoid of CPE exhibited significant cell death over a 2 week period in culture, while the neurons from WT littermates showed good survival. However, addition of CPE to the culture medium rescued these CPE-KO neurons from the cell death (Fig. 3). We also demonstrated that the neuroprotective effect of CPE does not depend on its enzymatic activity. CPE, in the presence of a specific inhibitor, GEMSA [25] (Fig. S2), or as an enzymatically inactive form of CPE (E300Q) [23], added as a recombinant protein to culture medium (data not shown), or from conditioned medium from cells expressing E300Q (Fig. 1C), all conferred neuroprotection on neurons subjected to oxidative stress suggesting that the CPE protein itself may confer this protection by binding to an interacting target molecule to initiate the signaling leading to cell survival. Work to identify this target is currently being pursued.

To investigate the signal transduction pathway for the neurotrophic function of CPE, we analyzed the effect of CPE on the activation of the MEK/ERK and PI3-K/AKT signaling pathways since both are major pathways for survival and neuroprotection [32]. Our study demonstrated that CPE increased ERK and AKT phosphorylation in primary cultured hippocampal neurons within 15 min of treatment. Moreover, the neuroprotection was blocked by ERK and AKT specific inhibitors. Interestingly, treatment with the AKT inhibitor (LY294004) or the ERK inhibitor (U0126) alone did not completely abolish the neuroprotective effect of CPE. However, it was completely abolished in the presence of both inhibitors, suggesting that both pathways contribute to the protection. These findings further support the function of CPE as a neurotrophic factor, exerting its neuroprotective effect by binding to a putative receptor, which in turn activates the ERK and AKT signaling pathways.

Hydrogen peroxide- and glutamate-induced neuronal death is due to production of reactive oxygen species (ROS) which cause cell death by apoptosis (versus necrosis). This is characterized by leakage of cytochrome c from the mitochondria, causing the activation of caspase 9 which in turn activates caspase-3 [33]. Our results show that treatment with CPE clearly prevented the activation of caspase-3 induced by hydrogen peroxide in the neurons (Fig. 6D). This data further support the survival and neuroprotective role of CPE in inhibiting apoptosis and suggest that the mechanism involves the recovery of mitochondrial energetics. The BCL-2 family is a large family of apoptosis regulator proteins. These include BCL-2 which is a pro-survival/anti-apoptotic protein and Bax which is a pro-apoptotic protein. Upon apoptosis signaling, Bax undergoes conformational changes which lead to its oligomerization and translocation into the mitochondrial membranes from the cytosol to form pores, leading to the release of cytochrome c and the activation of the cascade of caspases causing cell death [34], [35]. In contrast, the BCL-2 protein inhibits apoptosis-induced mitochondria pore formation to mediate cell survival [36]. Our data revealed that *Bcl-2* mRNA was up-regulated with CPE treatment (Fig. 6A) and the decrease in BCL-2 protein caused by oxidative stress was prevented by treatment with CPE (Fig. 6B and C). This observation is consistent with *Bcl-2* being a down-stream target gene of the AKT and ERK signaling pathways; pathways which can be activated by CPE in hippocampal neurons (Fig. 4).

The neuroprotective potency of CPE appears to be similar to BDNF. In the same experiment, CPE and BDNF provided the same extent of protection against oxidative stress within the same concentration range (Fig. S5A). Their mechanisms of action are also similar in that both activate the AKT and ERK pathways and increase BCL-2 expression to mediate neuroprotection [32], [37]. One possibility is that CPE might confer neuroprotection indirectly by up-regulating the expression of BDNF which in turn causes the increase in expression of BCL-2. However, that is not likely since treatment of hippocampal neurons with CPE did not change BDNF mRNA levels (Fig. S5B), and an inhibitor of the Trk receptor, K-252a, did not abolish the neuroprotective effect of CPE (Fig. S6). While it is possible that CPE’s function might be to up-regulate the expression of other growth factors, such as FGF2 or IGF1, reported to have neuroprotective and survival effects, studies have shown that the degree of activation of phosphorylation of AKT versus ERK required to mediate hippocampal neuroprotection by these two growth factors [37] differ from that of CPE. In those studies, IGF-1 showed very poor activation of AKT and ERK pathways compared to BDNF, while for FGF2, both pathways were activated, similar to BDNF, but only the AKT pathway played a role in the protection of hippocampal neurons upon apoptosis induced by low insulin in serum free medium. Additionally, we showed that an inhibitor of FGFR1, PD166285, did not attenuate the neuroprotective effect of CPE (Fig. S6). Hence, our findings support a direct role of CPE in up-regulating BCL-2 expression to mediate neuroprotection, rather than through the increase in expression of a brain derived neurotrophin, or a growth factor with neuroprotective properties.

Since we have now identified CPE as having neuroprotective properties, it is not surprising to find that CPE is associated with neurodegenerative diseases such as Alzheimer’s disease (AD). Indeed, a report demonstrated that in cortices from both AD patients and an AD mouse model, CPE was accumulated in dystrophic neurites surrounding amyloid plaques [38]. Given our new findings about CPE, we would hypothesize that the accumulated CPE in the neurites surrounding the plaques could be released as a defensive mechanism to protect the neurons against the amyloid beta toxicity. More interestingly, a CPE mutation found in the GeneBank EST database (dbEST) termed QQ CPE by us, was found to be from a patient with AD [39]. This QQ CPE mutant showed no secretion into the medium when transfected into AtT20 and Neuro2A cells, and when over-expressed in primary cultured cortical and hippocampal neurons, caused degeneration of the cells ultimately leading to cell death ([40] and Cawley *et al.*, manuscript in preparation). Such studies illustrate the potential importance of CPE as a neuroprotective factor in AD and other neurodegenerative diseases which is worthy of future investigations. In line with that, studies aiming at elucidating the role that CPE plays in the mouse model of neuronal ceroid lipofuscinoses in humans, where its expression is increased by >10 fold [31] can now be viewed in a different light. In addition, CPE synthesis is known to be up-regulated and presumably released from neurons to protect against neurodegeneration caused in some cases by increased circulating glucocorticoids after different types of stress [13], [14], [29].

**Figure 7:**
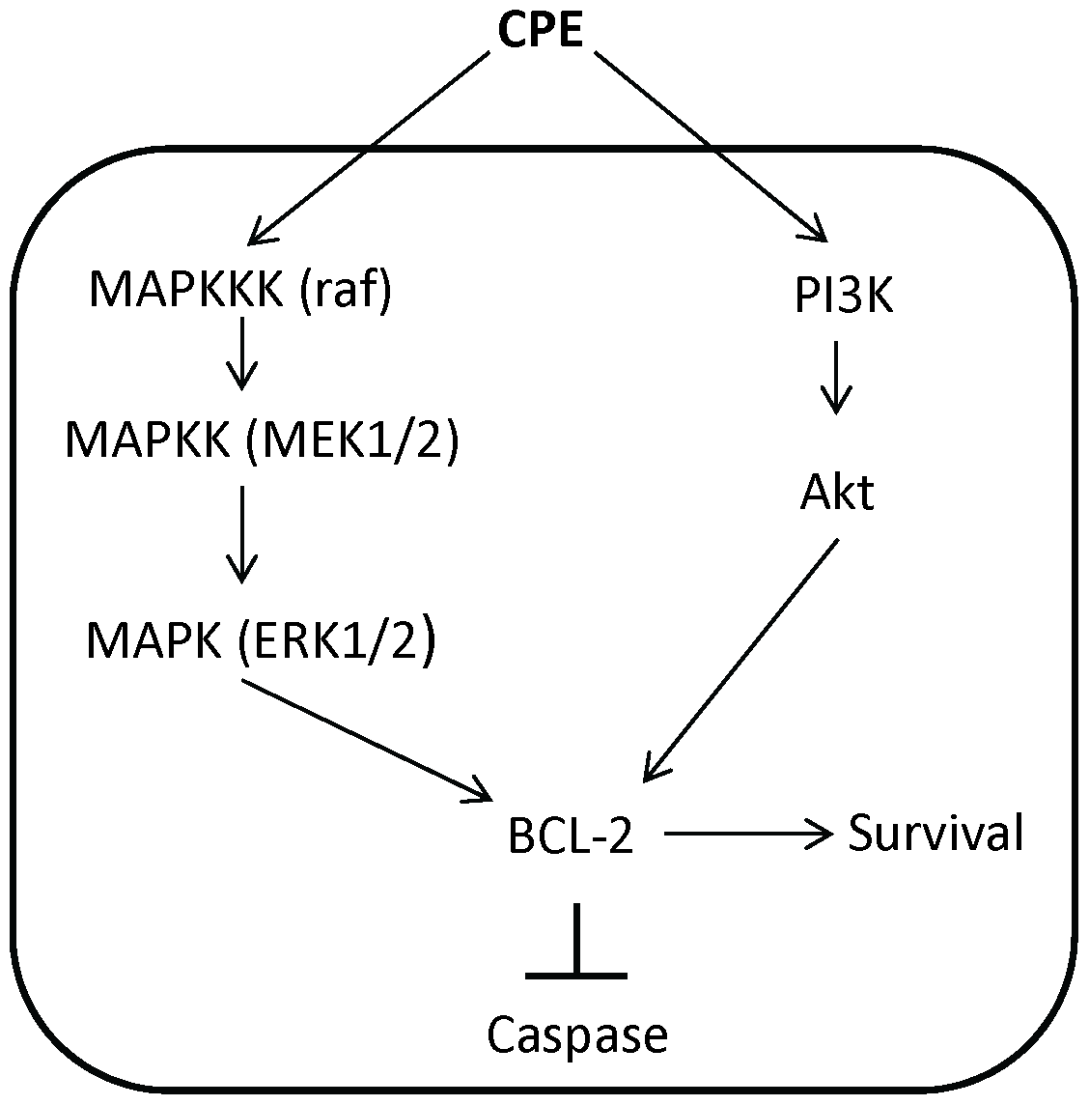
Mechanism of neuroprotection by CPE in primary cultured hippocampal neurons. CPE signals the MEK/ERK and PI3-K/AKT signaling pathways which up-regulate the expression of the anti-apoptotic protein, BCL-2, and inhibit the caspase cascade leading to a decrease in cleaved caspase-3, the apoptotic executioner.

In summary, our work demonstrates for the first time, the neuroprotective effects of secreted extracellular CPE, independent of its enzymatic activity, against different types of stress in neurons. These effects are mediated by MEK/ERK and PI3-K/AKT signaling pathways leading to increased expression of BCL-2, a pro-survival protein, to modulate mitochondrial energetics (Fig. 7). In addition, CPE inhibited activation of caspase-3, a major executor of apoptosis. We name this trophic factor “Neurotrophic Factor-α1” to distinguish CPE’s trophic role from its other previously described functions. Its activity can provide a new perspective for therapeutic interventions in neurodegenerative diseases.

## Supporting Information

Figure S1 **Characterization of recombinant CPE enzymatic activity.** Purified recombinant mCPE (20 ng and 60 ng) was incubated with 5 µg ACTH(1–17) in 50 mM sodium acetate, pH 5.5, 37°C, for 12 h. The specific CPE inhibitor, GEMSA, was used at 25 µM. The products generated were analyzed by high pressure liquid chromatography (HPLC). Briefly, the samples were separated by HPLC on a 4.6×250 mm 5 µm reverse phase Jupiter C18 column (Phenomenex, Torrance, CA), (Buffer A, 0.1% TFA; Buffer B, 80% Acetonitrile/0.1% TFA) and eluted with a 30%–34% Buffer B gradient over 12 min. The peptides were monitored by absorbance at 214 nm. Note that mCPE generated ACTH(1–16) and ACTH(1–15) in a dose dependent manner (20 ng, blue line; 60 ng, green line). All activity was prevented by the specific CPE inhibitor, GEMSA, (red line).
(PPTX)

Figure S2 **The neuroprotective effect of CPE are not dependent on its enzymatic activity.** To investigate if the enzymatic activity of CPE is required for it’s the neuroprotective effects, we used GEMSA, a specific enzyme inhibitor. Two µM GEMSA was added to CPE for 30 min before applying to primary cultured hippocampal neurons. **A)** Western blot analysis showing that CPE-induced phosphorylation of ERK and AKT was not affected by GEMSA. **B)** Bar graph shows that GEMSA did not inhibit the neuroprotective effect of CPE assessed by the WST-1 assay.
(PPTX)

Figure S3 **Neuroprotective effect of CPE on staurosporine and glutamate-induced neurotoxicity in primary cultured hippocampal neurons.** Bar graphs showing LDH activity in the culture media of hippocampal neurons treated with and without 0.2 µM Staurosporine (STS) (**A)** or 40 µM glutamate **(B)**. Note that the STS- and glutamate-induced cytotoxicity was significantly attenuated by the pretreatment of the neurons with 0.4 µM purified CPE. Students t test, n = 5, *** p<0.01 compared to control cells (Ctrl); ^###^ p<0.001 compared to treated only cells.
(PPTX)

Figure S4 **The neuroprotective effect of CPE on oxidative stress in rat cortical neurons.** Bar graph showing that cell viability of primary cultured rat E18 cortical neurons decreased significantly after 100 µM H_2_O_2_ treatment as assessed by the WST assay; however, pretreatment with CPE counteracted the H_2_O_2_-induced decrease in cell viability.
(PPTX)

Figure S5 **Effect of CPE and BDNF on neuroprotection and effect of CPE on BDNF mRNA expression. A)** Bar graph showing that CPE and BDNF had similar neuroprotective effects against H_2_O_2_-induced neurotoxicity as measured by the WST assay. **B)** Bar graph showing the quantification by qRT-PCR of *BDNF* mRNA in primary cultured hippocampal neurons after treatment with 0.4 µM CPE for 3 h. Data is normalized against 18S RNA and presented as a % compared to untreated control (Ctrl) cells.
(PPTX)

Figure S6 **Trk and FGF receptor inhibitors have no effect on CPE-mediated neuroprotection.** Bar graphs show that 1 µM of the Trk inhibitor, K-252a, or 1 µM of the FGFR1 inhibitor, PD166285, did not inhibit the neuroprotective effect of CPE against H_2_O_2_-induced neurotoxicity tested by the WST assay.
(PPTX)
