# Supplementary material for: Carboxypeptidase E/NFα1: A New Neurotrophic Factor against Oxidative Stress-Induced Apoptotic Cell Death Mediated by ERK and PI3-K/AKT Pathways
Source: PLoS One. 2013 Aug 15;8(8):e71578. doi: 10.1371/journal.pone.0071578 (PMC3744492; doi:10.1371/journal.pone.0071578)
Supplement: Figure S1 — Characterization of recombinant CPE enzymatic activity. Purified recombinant mCPE (20 ng and 60 ng) was incubated with 5 µg ACTH(1–17) in 50 mM sodium acetate, pH 5.5, 37°C, for 12 h. The specific CPE inhibitor, GEMSA, was used at 25 µM. The products generated were analyzed by high pressure liquid chromatography (HPLC). Briefly, the samples were separated by HPLC on a 4.6×250 mm 5 µm reverse phase Jupiter C18 column (Phenomenex, Torrance, CA), (Buffer A, 0.1% TFA; Buffer B, 80% Acetonitrile/0.1% TFA) and eluted with a 30%–34% Buffer B gradient over 12 min. The peptides were monitored by absorbance at 214 nm. Note that mCPE generated ACTH(1–16) and ACTH(1–15) in a dose dependent manner (20 ng, blue line; 60 ng, green line). All activity was prevented by the specific CPE inhibitor, GEMSA, (red line). (PPTX) [file pone.0071578.s001.pptx]

## Slide 1
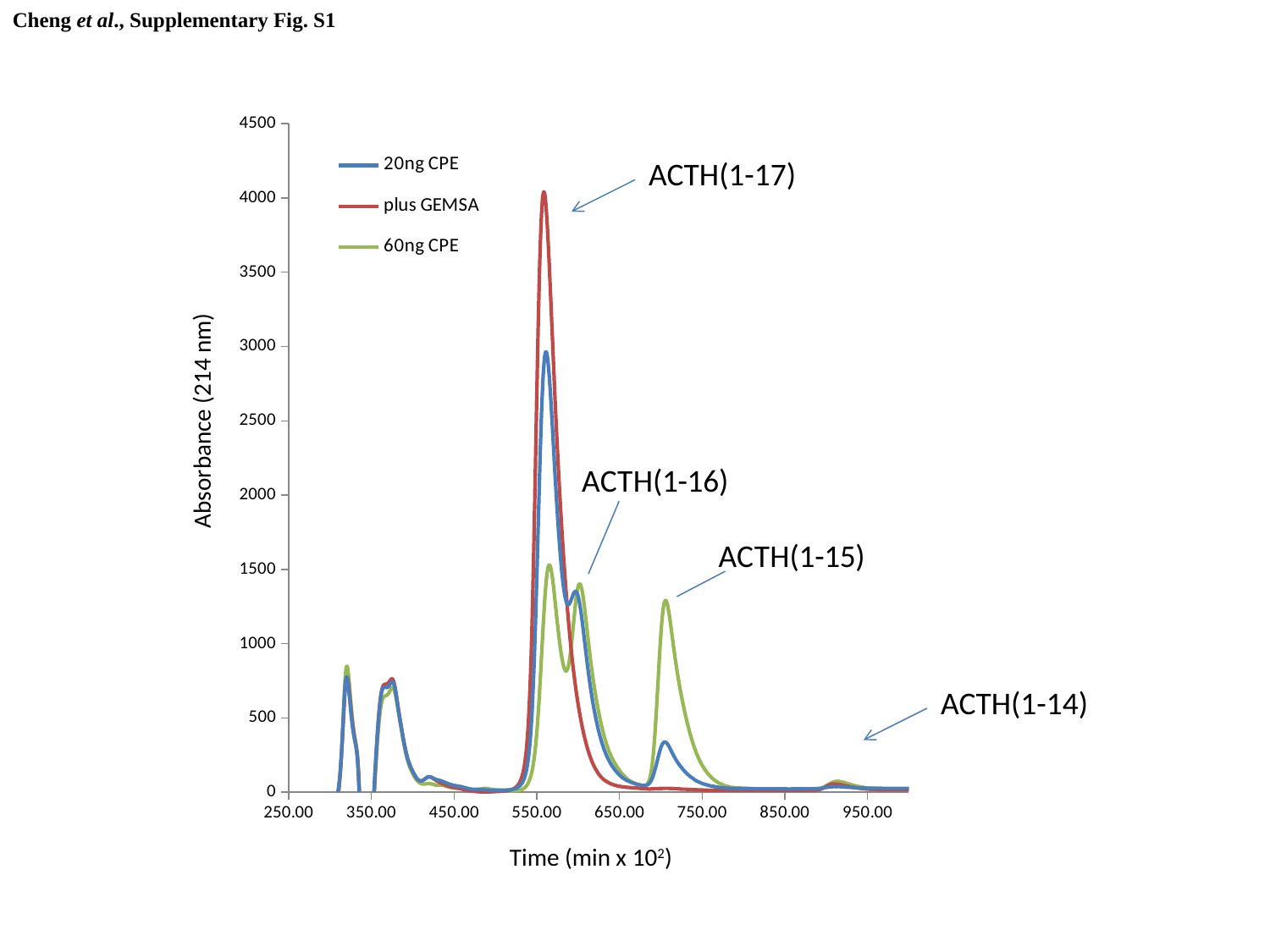

Cheng et al., Supplementary Fig. S1
### Chart
| Category | 20ng CPE | plus GEMSA | 60ng CPE |
|---|---|---|---|ACTH(1-17)
Absorbance (214 nm)
ACTH(1-14)
Time (min x 102)
