# Supplementary figures and images for: Carboxypeptidase E/NFα1: A New Neurotrophic Factor against Oxidative Stress-Induced Apoptotic Cell Death Mediated by ERK and PI3-K/AKT Pathways
Source: PLoS One. 2013 Aug 15;8(8):e71578. doi: 10.1371/journal.pone.0071578 (PMC3744492; doi:10.1371/journal.pone.0071578)

## Slide 1
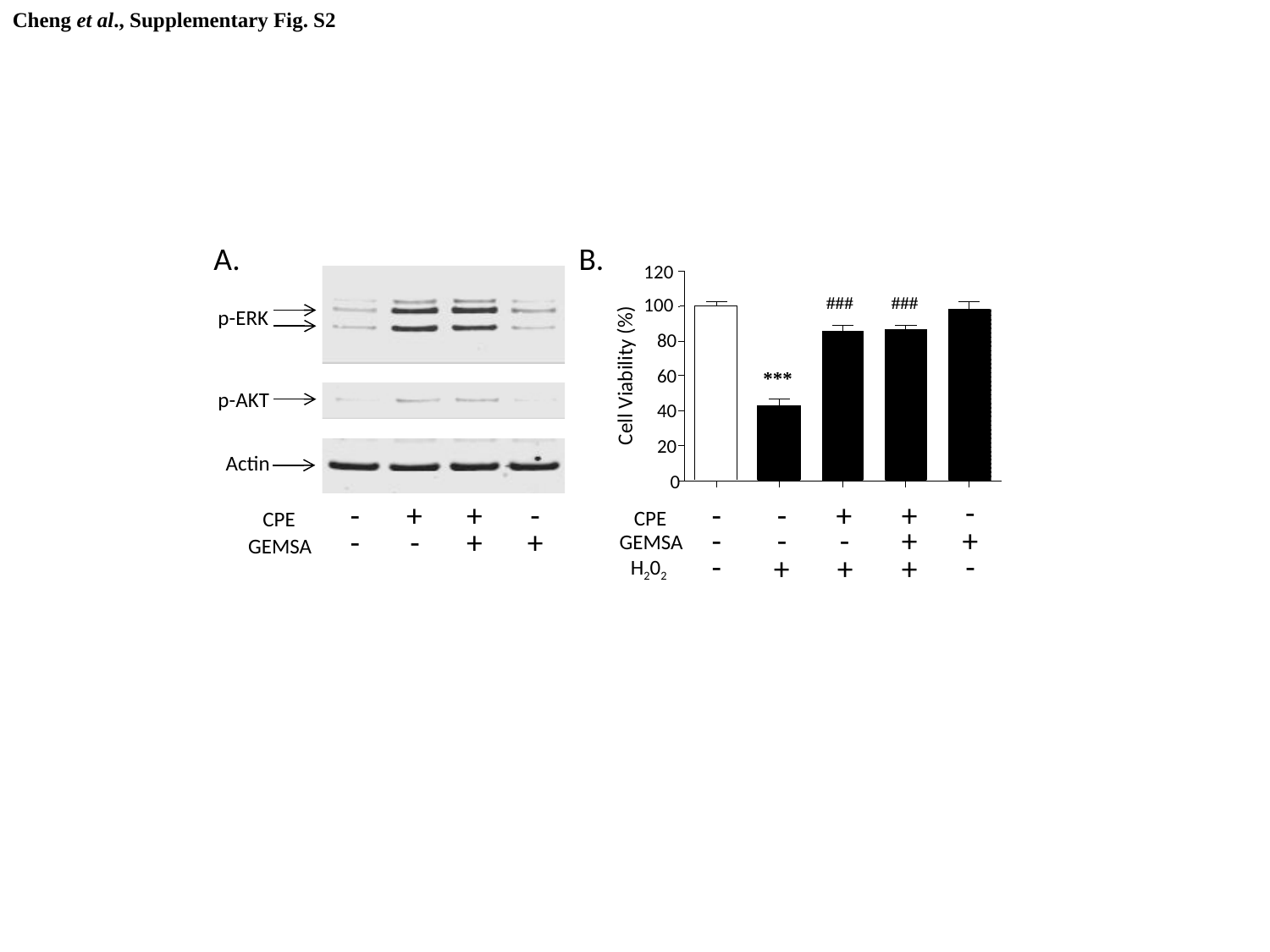

Cheng et al., Supplementary Fig. S2
A.
B.
120
###
###
100
80
Cell Viability (%)
60
***
40
20
0
-
-
+
+
-
CPE
-
-
+
+
-
GEMSA
-
-
+
+
+
H202
p-ERK
p-AKT
Actin
-
+
+
-
CPE
-
-
+
+
GEMSA

Supplement: Figure S2 — The neuroprotective effect of CPE are not dependent on its enzymatic activity. To investigate if the enzymatic activity of CPE is required for it’s the neuroprotective effects, we used GEMSA, a specific enzyme inhibitor. Two µM GEMSA was added to CPE for 30 min before applying to primary cultured hippocampal neurons. A) Western blot analysis showing that CPE-induced phosphorylation of ERK and AKT was not affected by GEMSA. B) Bar graph shows that GEMSA did not inhibit the neuroprotective effect of CPE assessed by the WST-1 assay. (PPTX) [file pone.0071578.s002.pptx]

## Slide 1
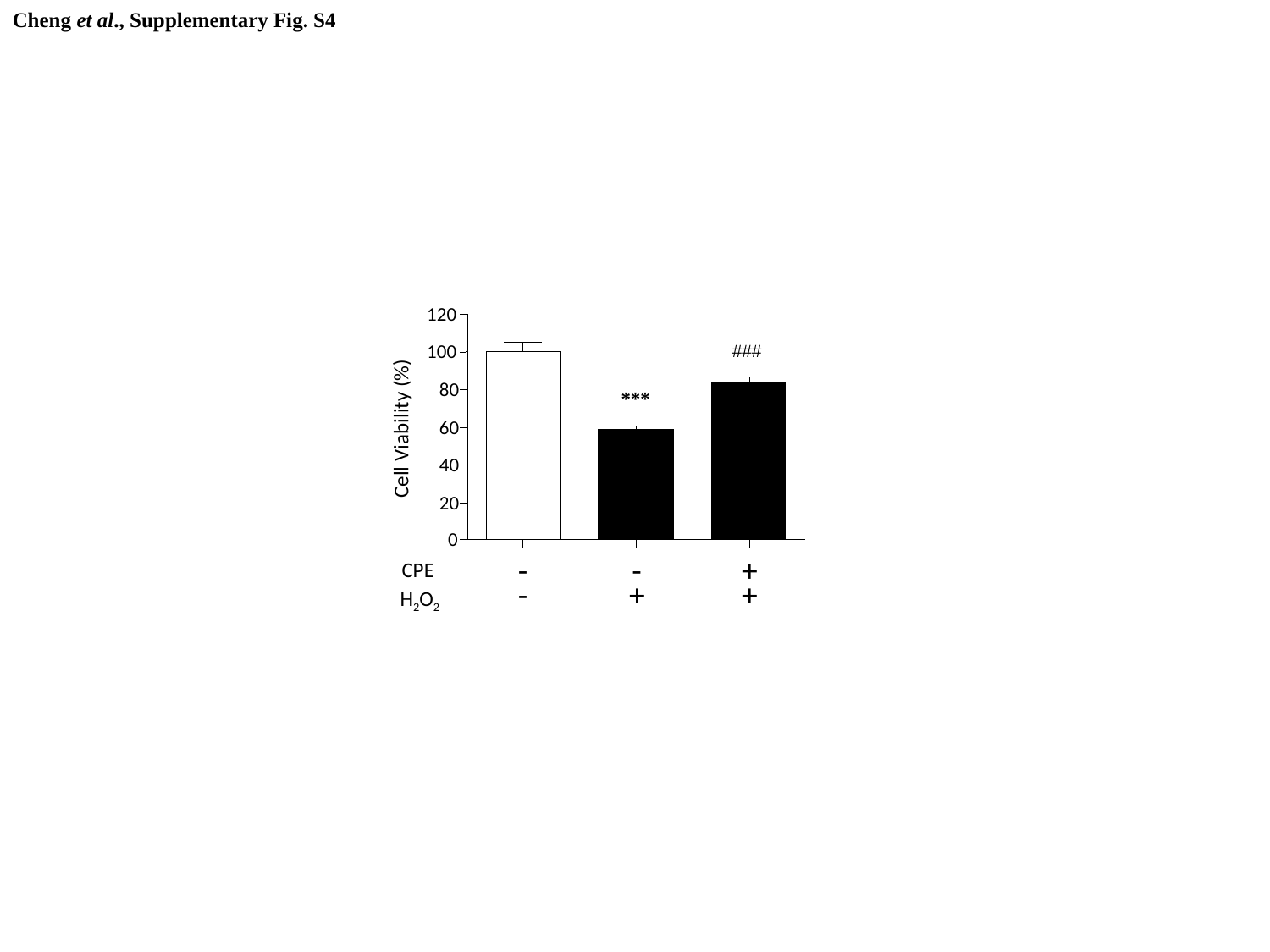

Cheng et al., Supplementary Fig. S4
120
100
###
80
***
60
Cell Viability (%)
40
20
0
-
-
+
CPE
-
+
+
H2O2

Supplement: Figure S4 — The neuroprotective effect of CPE on oxidative stress in rat cortical neurons. Bar graph showing that cell viability of primary cultured rat E18 cortical neurons decreased significantly after 100 µM H2O2 treatment as assessed by the WST assay; however, pretreatment with CPE counteracted the H2O2-induced decrease in cell viability. (PPTX) [file pone.0071578.s004.pptx]

## Slide 1
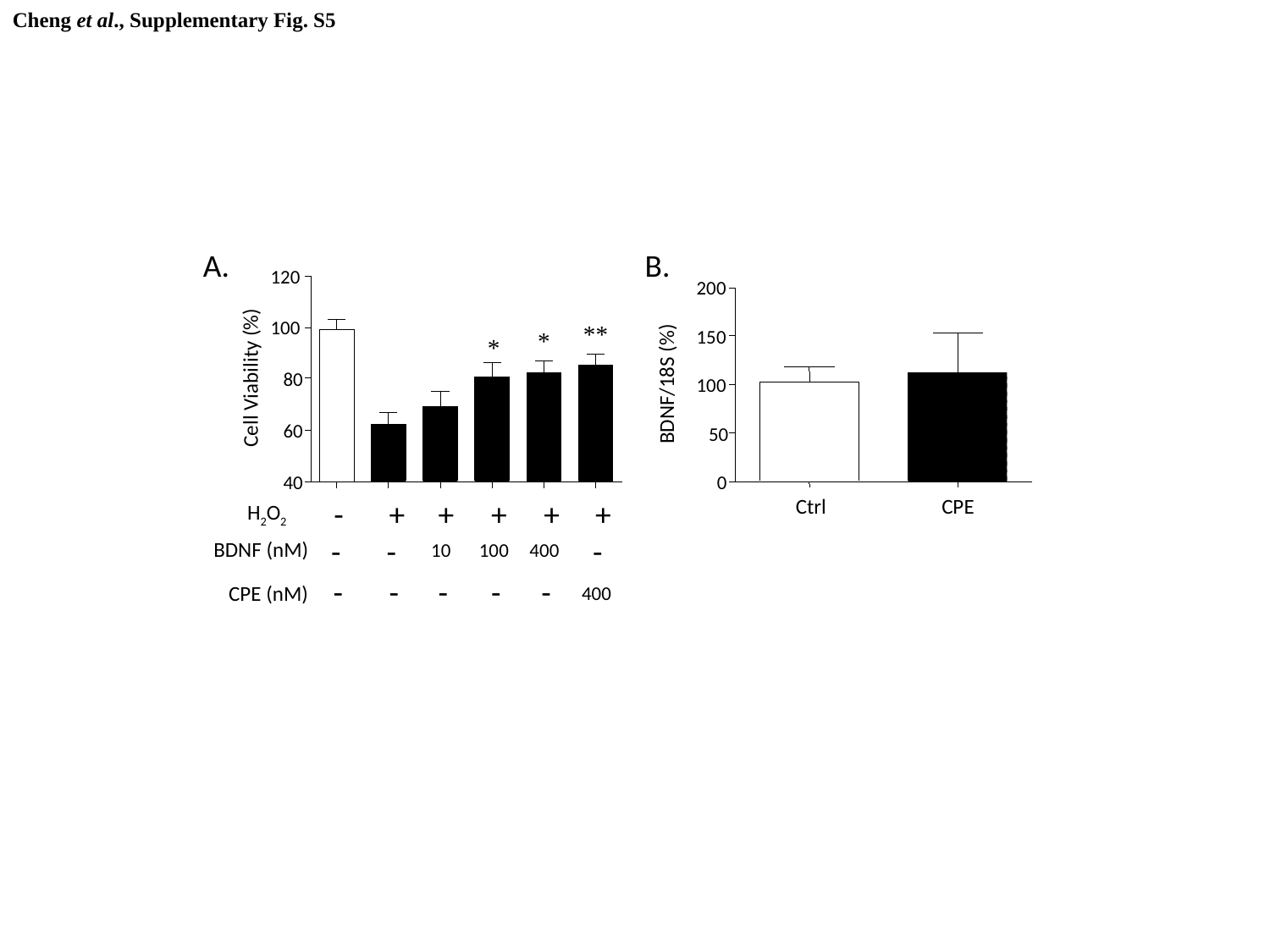

Cheng et al., Supplementary Fig. S5
A.
B.
120
100
Cell Viability (%)
80
60
40
-
+
+
+
+
+
H2O2
-
-
-
BDNF (nM)
10
100
400
-
-
-
-
-
CPE (nM)
400
200
150
BDNF/18S (%)
100
50
0
Ctrl
CPE
**
*
*

Supplement: Figure S5 — Effect of CPE and BDNF on neuroprotection and effect of CPE on BDNF mRNA expression. A) Bar graph showing that CPE and BDNF had similar neuroprotective effects against H2O2-induced neurotoxicity as measured by the WST assay. B) Bar graph showing the quantification by qRT-PCR of BDNF mRNA in primary cultured hippocampal neurons after treatment with 0.4 µM CPE for 3 h. Data is normalized against 18S RNA and presented as a % compared to untreated control (Ctrl) cells. (PPTX) [file pone.0071578.s005.pptx]

## Slide 1
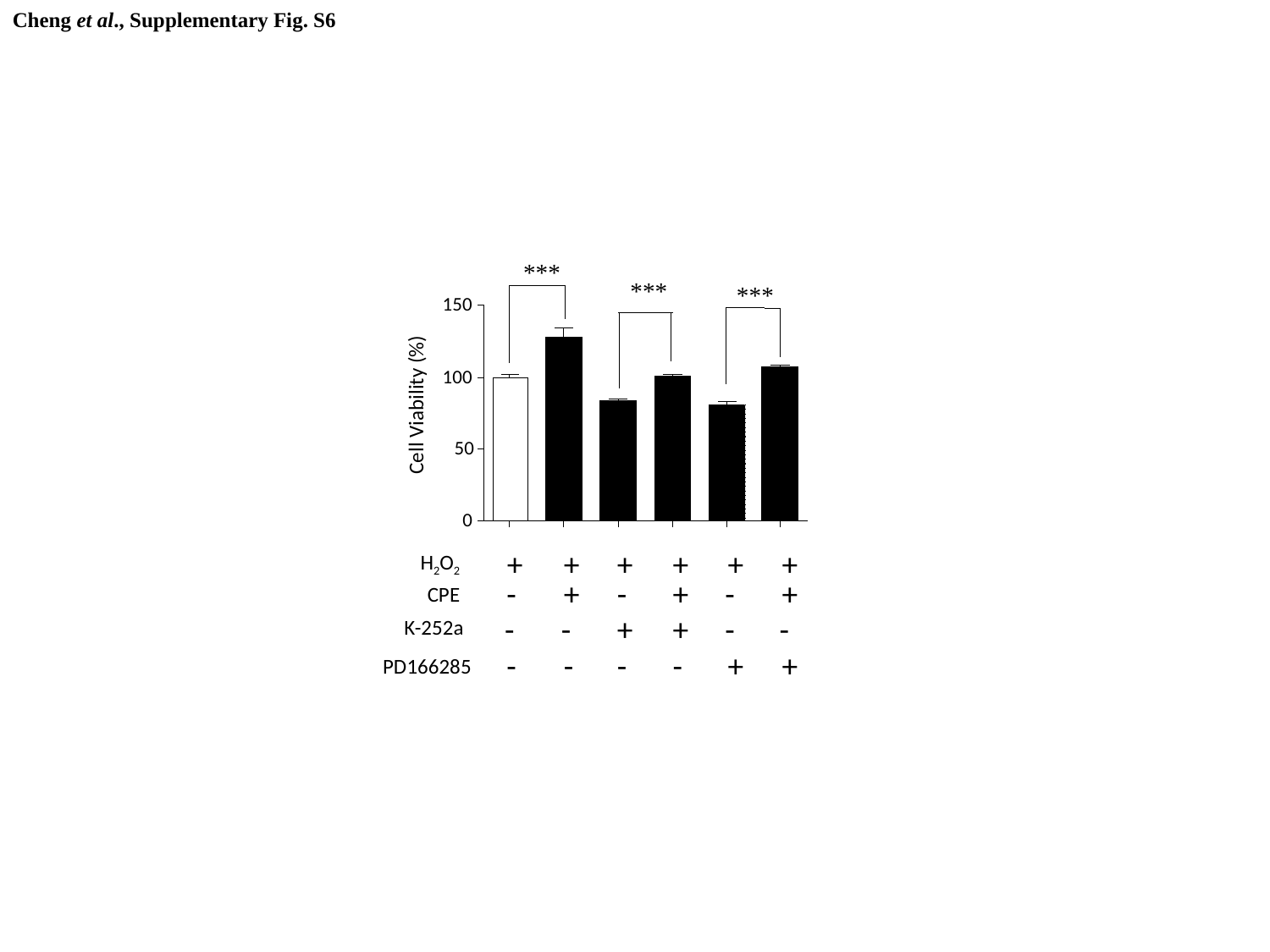

Cheng et al., Supplementary Fig. S6
***
***
***
150
100
Cell Viability (%)
50
0
+
+
+
+
+
+
H2O2
-
+
-
+
-
+
CPE
-
-
+
+
-
-
K-252a
-
-
-
-
+
+
PD166285

Supplement: Figure S6 — Trk and FGF receptor inhibitors have no effect on CPE-mediated neuroprotection. Bar graphs show that 1 µM of the Trk inhibitor, K-252a, or 1 µM of the FGFR1 inhibitor, PD166285, did not inhibit the neuroprotective effect of CPE against H2O2-induced neurotoxicity tested by the WST assay. (PPTX) [file pone.0071578.s006.pptx]
