# Supplementary material for: Carboxypeptidase E/NFα1: A New Neurotrophic Factor against Oxidative Stress-Induced Apoptotic Cell Death Mediated by ERK and PI3-K/AKT Pathways
Source: PLoS One. 2013 Aug 15;8(8):e71578. doi: 10.1371/journal.pone.0071578 (PMC3744492; doi:10.1371/journal.pone.0071578)
Supplement: Figure S3 — Neuroprotective effect of CPE on staurosporine and glutamate-induced neurotoxicity in primary cultured hippocampal neurons. Bar graphs showing LDH activity in the culture media of hippocampal neurons treated with and without 0.2 µM Staurosporine (STS) (A) or 40 µM glutamate (B). Note that the STS- and glutamate-induced cytotoxicity was significantly attenuated by the pretreatment of the neurons with 0.4 µM purified CPE. Students t test, n = 5, *** p<0.01 compared to control cells (Ctrl); ### p<0.001 compared to treated only cells. (PPTX) [file pone.0071578.s003.pptx]

## Slide 1
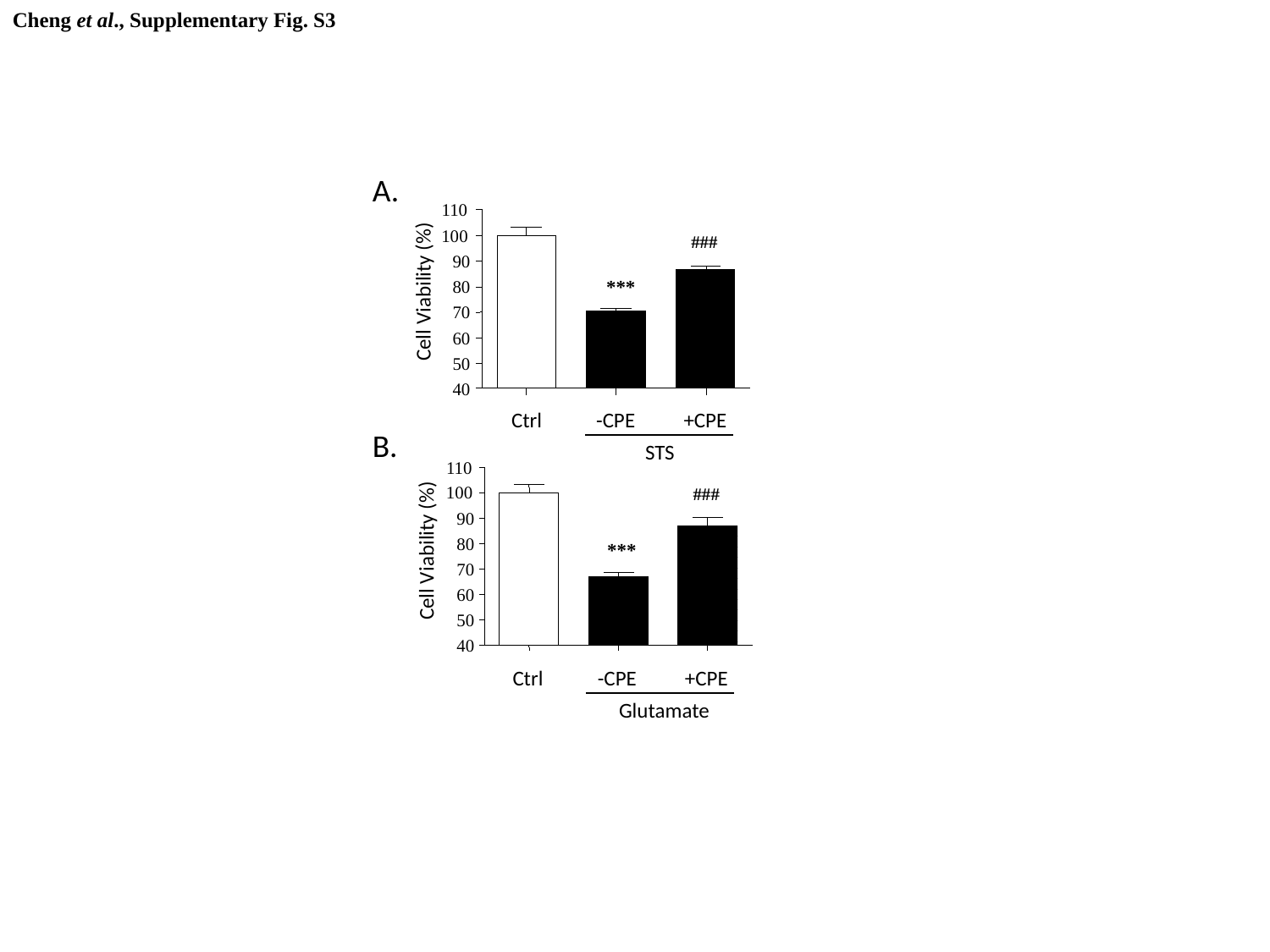

Cheng et al., Supplementary Fig. S3
A.
110
100
###
90
***
80
 Cell Viability (%)
70
60
50
40
Ctrl
-CPE
+CPE
STS
B.
110
100
###
90
80
***
 Cell Viability (%)
70
60
50
40
Ctrl
-CPE
+CPE
Glutamate
